# Exceptional appendage and soft-tissue preservation in a Middle Triassic horseshoe crab from SW China

**DOI:** 10.1038/s41598-017-13319-x

**Published:** 2017-10-26

**Authors:** Shixue Hu, Qiyue Zhang, Rodney M. Feldmann, Michael J. Benton, Carrie E. Schweitzer, Jinyuan Huang, Wen Wen, Changyong Zhou, Tao Xie, Tao Lü, Shuigen Hong

**Affiliations:** 1 Chengdu Center, China Geological Survey, Chengdu, 610081 China; 2 Chengdu Insitute of Geology and Mineral Resources, Chengdu, 610081 China; 3 Department of Geology, Kent State University, Kent, OH 44242 USA; 4 School of Earth Sciences, University of Bristol, Bristol, BS8 1RJ UK; 5 Department of Geology, Kent State University at Stark, 6000 Frank Avenue NW, North Canton, OH 44720 USA; 6 Institute of Neuroscience, Xiamen University, Xiamen, 361005 China

## Abstract

Horseshoe crabs are classic “living fossils”, supposedly slowly evolving, conservative taxa, with a long fossil record back to the Ordovician. The evolution of their exoskeleton is well documented by fossils, but appendage and soft-tissue preservation is extremely rare. Here we analyse details of appendage and soft-tissue preservation in *Yunnanolimulus luopingensis*, a Middle Triassic (ca. 244 million years old) horseshoe crab from Yunnan Province, SW China. The remarkable preservation of anatomical details including the chelicerae, five pairs of walking appendages, opisthosomal appendages with book gills, muscles, and fine setae permits comparison with extant horseshoe crabs. The close anatomical similarity between the Middle Triassic horseshoe crabs and their recent analogues documents anatomical conservatism for over 240 million years, suggesting persistence of lifestyle. The occurrence of *Carcinoscorpius*-type claspers on the first and second walking legs in male individuals of *Y. luopingensis* indicates that simple chelate claspers in males are plesiomorphic for horseshoe crabs, and the bulbous claspers in *Tachypleus* and *Limulus* are derived.

## Introduction

Horseshoe crabs are marine invertebrates well known as examples of evolutionary conservatism^1,2^. They have a sparse fossil record, with only about 30 fossil genera known^3–6^. The known data indicate that the earliest fossil representatives of Limulidae, the family that includes all extant and most Mesozoic taxa, probably appeared in the Triassic^7,8^. Preservation of the soft tissues in fossil horseshoe crabs is rare and their anatomical details are still poorly known. Previous reports on preservation of appendages and soft tissues are mainly from Palaeozoic taxa, especially synziphosurines^9–13^. However, there is increasing evidence that synziphosurines are not Xiphosura at all but rather stem euchelicerates^7,8,14,15^. Within the class of Xiphosura, preservation of appendages in fossil taxa includes *Euproops* ^16^, *Alanops magnificus* ^17^, *Paleolimulus signatus* ^4^, *Psammolimulus gottingensis* ^18^, *Victalimulus mcqueeni* ^19^, and *Tachypleus syriacus* ^20^. Preservation of muscles in fossil horseshoe crabs is even more rare^20,21^. Thus any new information about appendages and soft-tissues of fossil horseshoe crabs would shed new light on understanding their evolution.

Recently, a Middle Triassic horseshoe crab, *Yunnanolimulus luopingensis*, was recovered from a Middle Triassic fossil lagerstätte in Luoping, Yunnan, China^22^, representing the first record of horseshoe crabs from China and the eastern Tethys. Later, *Y. luopingensis* was assigned to the Limulidae^5,8^. Further preparation of the described specimens and newly collected specimens have led to the discovery of well preserved anatomical details, including book gills, pusher legs, muscles, and setae.

The horseshoe crab fossils were collected from the finely laminated micritic limestone of the middle part of Member II of the Guanling Formation around Dawazi Village, Luoping Town, Yunnan Province, China. From the same interval, associated with *Y. luopingensis* are marine reptiles, fishes, arthropods, bivalves, gastropods, echinoderms, brachiopods, conodonts, foraminifers, ammonoids, belemnoids, and lingulid brachiopods, as well as a few terrestrial millipedes and conifer plants^22,23^. The age of the Luoping biota is constrained to the Pelsonian substage (ca. 244 million years), Anisian, Middle Triassic by the index conodont *Nicoraella kockeli* ^24^. The diversity of the Luoping biota makes it one of the most important Triassic fossil lagerstätten in terms of understanding the recovery and radiation of marine ecosystems after the profound Permo-Triassic mass extinction^23,25^. The exceptional preservation of associated fossils has been interpreted as caused by anoxia of the bottom water and microbial sealing^23,26^. It can be inferred that the horseshoe crabs suffered a similar fate.

**Figure 1:**
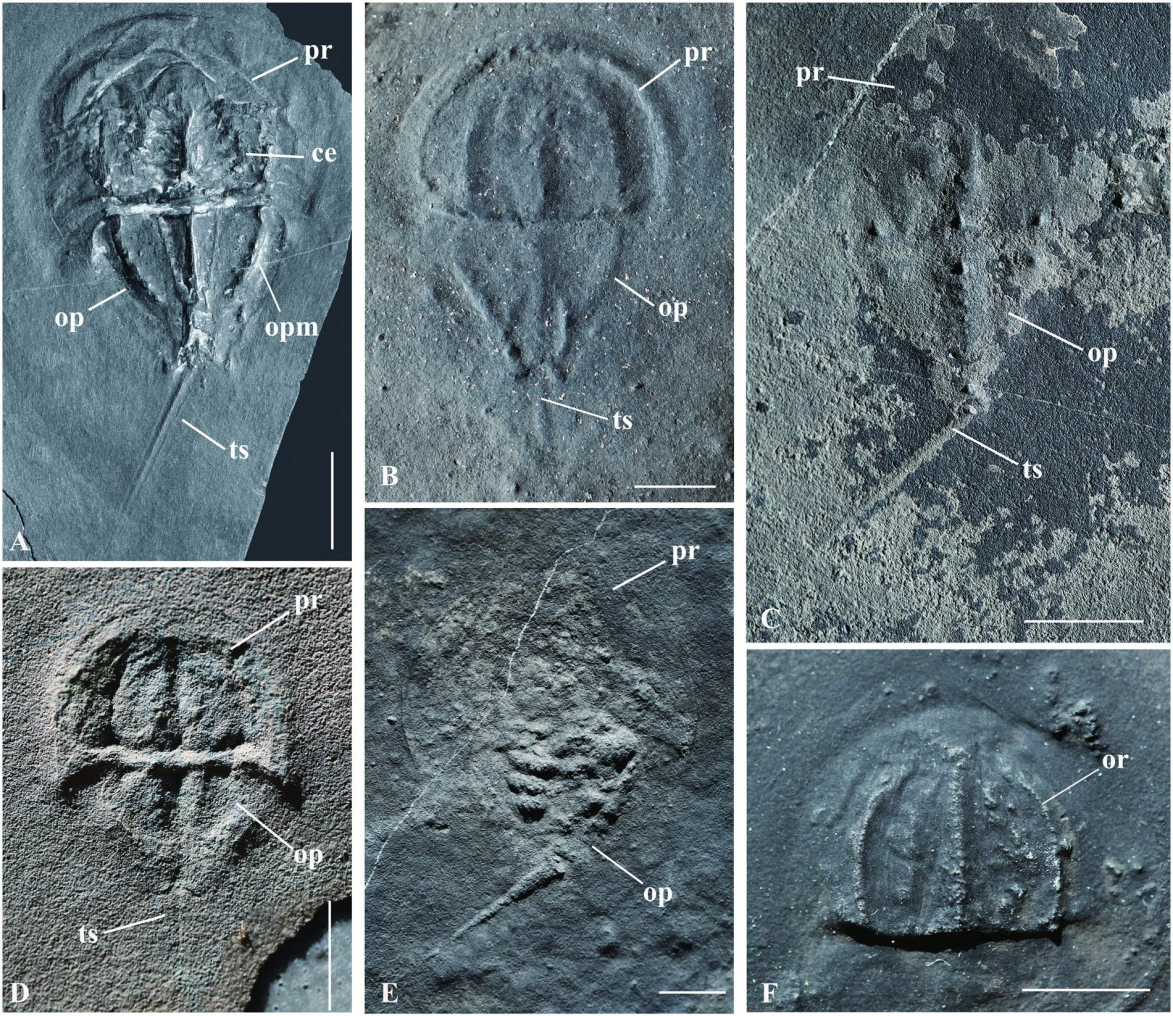
The Middle Triassic horseshoe crab *Yunnanolimulus luopingensis*. (**A**) Holotype, LPI-61299; (**B**) LPI-31908; (**C**) LPI-32169; (**D**) LPI-31910; an external mold; (**E**) LPI-32185, ventral view; (**F**) LPI-31926, only the prosoma preserved; The following abbreviations are used: ce, compound eyes; op, opisthosoma; opm, opisthosomal margin; or, ophthalmic ridges; pr, prosoma; ts, telson. Scale bars: 10 mm in (**A**) and (**C**); 5 mm in (**B**) and (**D**–**F**).

**Figure 2:**
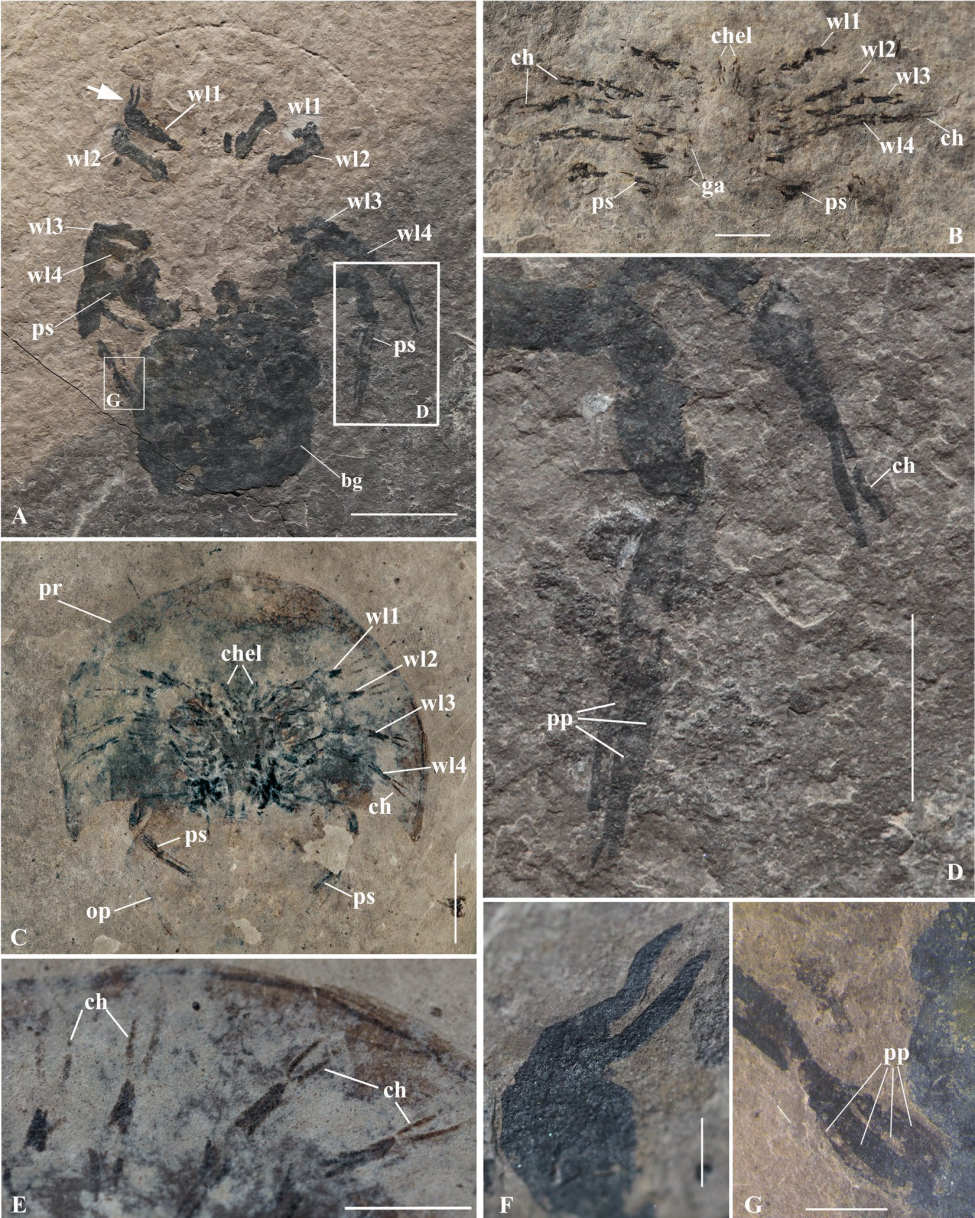
Prosomal appendages of *Yunnanolimulus luopingensis*. (**A**) LPI-61734, a well-preserved individual with walking legs and book gills in ventral view. Frame indicates close-up shown in (**D**,**G**). **(B**) LPI-3630, a juvenile individual with well-preserved prosomal appendages. (**C**) LPI-38878, a juvenile individual with preserved prosomal appendages; (**D**) details of the pusher leg and the chela of 5th right walking leg, note the whorl of plates and the smaller chela on the pusher. (**E**) Enlargement of the 2–5 right pairs of walking legs in (**C**). (**F**) Enlargement of the details of the first left walking legs in (**A**) (indicated by the white arrow); (**G**) details of the plates on the left pusher in (**A**). The following abbreviations are used: bg, book gills; ch, chela; chel, chelicerae; ga, gnathobase; ms, movable spine; op, opisthosoma; pp, pusher plates; pr, prosoma; ps, pusher; wl, walking leg. Scale bars: 10 mm in (**A**); 2 mm in B; 5 mm in (**C**,**D**); 2 mm in E; 1 mm in (**F**,**G**).

**Figure 3:**
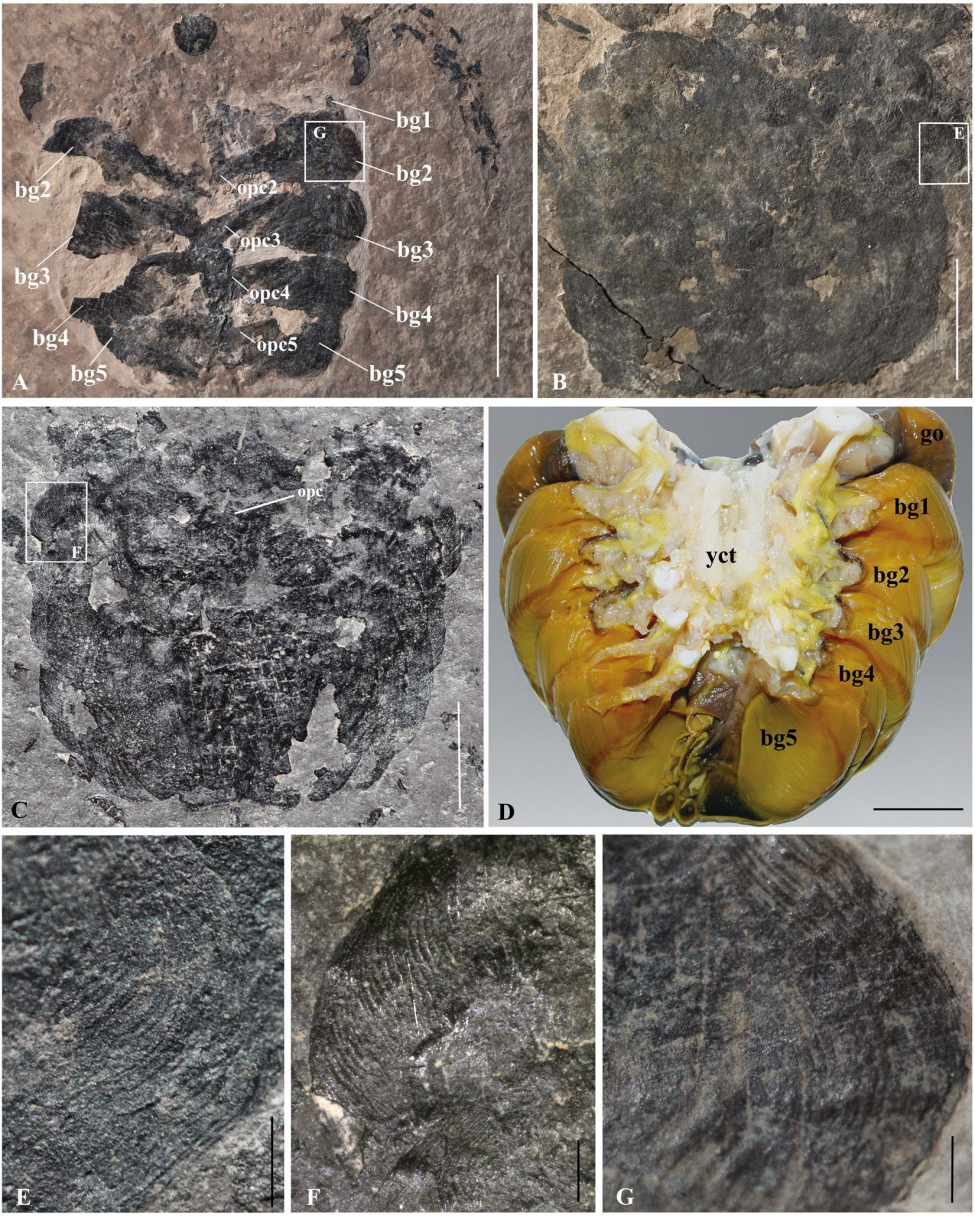
Book gills of *Yunnanolimulus luopingensis* and extant horseshoe crabs. (**A**) LPI-60564, an individual with preserved book gills and pusher legs; frame indicates close-up shown in (**G**). (**B**) LPI-61734, enlargement of opisthosomal part with book gills; frame indicates close-up shown in (**E**). (**C**) LPI-40709, showing the book gills and movable spines; frame indicates close-up shown in (**F**). (**D**) Five pairs of book gills of the extant *Tachypleus tridentatus*, note the operculum was removed. Provided by Shuigen Hong; (**E**), (**F**), (**G**) enlargement of frames in (**B**), (**C**), (**A**) respectively, showing details of respiratory lamellae. The following abbreviations are used: opc2-opc5, second to fifth gill opecula; bg, book gills; ms, movable spines; opc: genital operculum; yct, yellow connective tissue. Scale bars: 10 mm in A; 5 mm in (**A**–**C**); 2 cm in **D**; 500 μm in (**E**–**G**).

**Figure 4:**
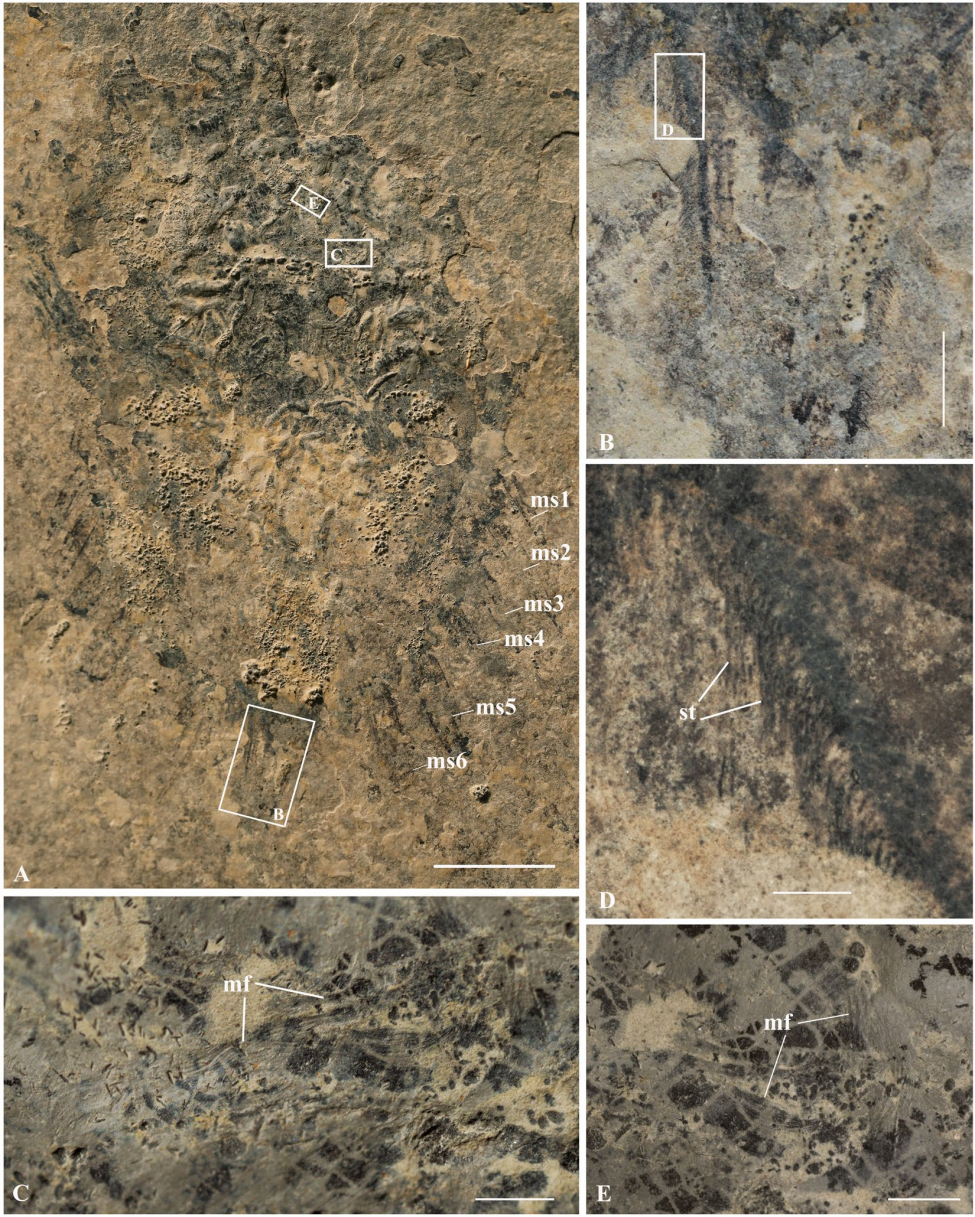
*Yunnanolimulus luopingensis* with preserved setae and muscles. (**A**) LPI-31945, a large individual. Frames indicate close-ups shown in (**B**), (**C**), and (**E**). (**C**,**E**) details of muscles, showing the fiberes. (**B**), (**D**) details of setae on two sides of the telson. (**D**) Is the enlargement of the frame in (**B**). The following abbreviations are used: mf, muscle fibre; ms, movable spines; st, setae. Scale bars: 10 mm in (**A**); 1 mm in (**B**) and (**C**); 500 μm in (**D**) and (**E**).

A total of 12 individuals is available for study, of which six specimens preserve only exoskeletal parts (Fig. 1). Well preserved prosomal appendages are observed in an adult (LPI-61734) and three juvenile individuals (LPI-3630, LPI-38878, LPI-60564) (Fig. 2), and book gills are seen in three individuals (LPI-40709,  LPI-60564, LPI-61734) (Fig. 3). Preservation of muscles and setae is recognized from a single large individual (LPI-31945) (Fig. 4). Based upon the close association of all the specimens and no evidence to the contrary, we regard them as members of the same species. It is worth noting that at the time of original description of the species^22^, prosomal appendages were mentioned, but not discussed in detail. The details of pusher legs were revealed by further preparation of the described specimens (LPI-61734).

## Results

**Systematic Palaeontology**

Order XIPHOSURIDA Latreille, 1802 (=MEROSTOMATA Dana, 1852)

Suborder LIMULINA Richter and Richter, 1929

Superfamily LIMULOIDEA Zittel, 1885

Family LIMULIDAE Zittel, 1885

Genus *Yunnanolimulus* Zhang, Hu, Zhou, Lü,  and Bai, 2009

*Yunnanolimulus luopingensis* Zhang, Hu, Zhou, Lü,  and Bai, 2009

### Revised diagnosis

Prosoma gently vaulted, semi-circular in outline, wider than long, lateral sides parallel, distally continuous into two genal angles. Cardiac lobe about 3/5 width of prosoma, tapering gradually forward. Ophthalmic ridges distinct, not meeting in front of the cardiac lobe. Eyes are low. Genal spines triangular, posteriorly directed, forming an acute angle with anterolateral margin of opisthosoma. Opisthosoma subtriangular, non-segmented, slightly wider than cardiac lobe, tapering backward gradually. Hinge relatively straight. Axial region in opisthosoma distinct, with a width 1/3 that of the opisthosoma. Six pairs of movable spines present on both sides of opisthosoma. Telson long and sword-like in outline, triangular in cross-section, with length equal to the body.

### Remarks

*Yunnanolimulus luopingensis* was originally assigned to the family Mesolimulidae^22^, but, as indicated by later works^5,8^, it should be moved to the Family Limulidae. Among the nine described genera of Triassic horseshoe crabs^27^,*Yunnanolimulus* is similar to some species of *Limulitella* in general. Recent work has proved that *Limulitella* is a polyphyletic genus^8^, thus, a comparision at the species level is more reliable. Both *Y. luopingensis* and *Limulitella bronni* show a similar angle between the genal spines and the opisthosoma, but the ophisthosoma of *Y. luopingensis* is subtriangular, whereas it is round in *L. bronni*. The prosoma/opisthosoma ratio of *Y. luopingensis* is about 1:1, but 1:0.8 in *L. bronni*. Compared to *Limulitella tejraensis* ^27^, *Y. luopingensis* has a relatively shorter opisthosoma and longer movable spines. The outward extension of the genal spines, and the two longer posterior marginal spines in *Pasmmolimulus gottingensis* ^18^ make it different from *Y. luopingensis*. It also differs from the Palaeozoic *Palaeolimulus signatus* ^4^ in the more posterior compound eyes, the posterior placement of the prosomal appendages and the mouth, and the ophthalmic ridges not meeting in the front. *Yunnanolimulus luopingensis* differs from *Mesolimulus walchi* in the broader pleura and the parallel ophthalmic ridges on the prosoma of the latter.

### Occurrence

Anisian, Middle Triassic. All specimens were recovered from the middle part of Member II of the Guanling Formation in Dawazi and neighbouring areas in Luoping County, Yunnan Province, SW China. The GPS position of the fossil locality is 103°44′54″N, 69°56′9″E.

### Description

The body is composed of three parts: a prosomal shield, fused opisthosomal tergites, and a styliform telson (Fig. 1A–E). The maximum length of the combined prosoma and opisthosoma is 40 mm in the largest individual (LPI-31945), whereas the minimum length is 15 mm in the smallest individual (LPI-31910).

The prosoma is hemispherical in outline, with a maximum width of 10.4 mm. The two ophthalmic ridges are distinct, but never meet at the front. The area between the two ophthalmic ridges is relatively wide posteriorly, about 3/5 the width of the prosoma (Fig. 1B,E). Anteriorly, the width of the area decreases to about 1/2 the prosomal width. The compound eyes are located on the posterior 1/3 of the ophthalmic ridges. The two simple eyes (ocelli) are not preserved. The interophthalmic region is U-shaped and parallel-sided posteriorly, converging anteriorly and becoming obscure or indistinct anteriorly. The two sides of the posterolateral corners of the prosoma extend posteriorly and slightly axially, forming two broad-based, acutely pointed genal spines.

There are three marginal tubercles of equal size in the posterior part of the axial region (Fig. 1C): a median tubercle and two symmetrical tubercles positioned on the posterior end of the ophthalmic ridges. Whether the distal parts of the tubercles are pointed is not clear. Two upward pointed spines are present on the prosoma of the extant *Tachypleus tridentatus* ^17^ in the same position as those on *Yunnanolimulus luopingensis*. The posterior margin of the prosoma is nearly straight, but curving posterolaterally at the genal spines.

The opisthosoma is a truncated triangle in outline (Fig. 1D). The anterior margin is parallel to the posterior margin of the prosoma. The width of the opisthosoma is nearly the same as the posterior part of the interophthalmic region on the prosoma. Three marginal tubercles are present on the anterior margin of the opisthosoma, one in the midline and the other two on either side of the axis (Fig. 1C), corresponding to the positions of the three spines on the prosoma. Axial furrows on the opisthosoma are distinct in the holotype (Fig. 1A), but less distinct or even invisible in other specimens. This difference may be the result of taphonomic biases. Four pairs of nodes/tubercles are situated on the ridge separating the vaulted axial region and the opisthosomal rim. The opisthosomal rim is broad and moderately flat. The lateral margins of the opisthosoma converge posteriorly to two broad, triangular, immovable spines, forming a concavity to enclose the telson.

There are six pairs of long, slender, movable spines, increasing in size posteriorly on the lateral margins of the opisthosoma. The movable spines articulate within concave sockets. The intercalated fixed spines are short and triangular. In the largest individual (LPI-31945, Fig. 4A), the length of the second pair of movable spines is about 10 mm, and the width at the base is 1.5 mm, whereas the preserved length of the last pair is 15 mm, and the width at the base is 2.5 mm. Given that the specimen is the largest individual of *Y. luopingensis*, most probably it represents a mature adult rather than a juvenile. Therefore, the posterior increase of movable spine length in *Y. luopingensis* could be the result of sexual dimophism. In extant horseshoe crabs, male *Tachypleus tridentatus* shows a posterior increase of movable spines (Supplementary Fig. S1). In *Tachypleus* and *Limulus*, the marginal spines also exhibit sexual dimorphism, with the posterior three pairs being extremely short in mature females.

*Yunnanolimulus luopingensis* bears a long, styliform telson, as long as the combined sagittal length of the prosoma and opisthosoma, or even longer. In dorsal view, the outline of the telson is lanceolate, with a median keel (Fig. 1A). The width of the telson decreases distally. The underside of the telson is not preserved in most of the specimens. Only one specimen, LPI-32185 (Fig. 1E), exhibits a recurved underside. The underside of the telson is tentatively interpreted as recurved herein, but more evidence is needed to clarify this statement.

### Ventral anatomy and soft tissues

Prosomal appendages are observed in three specimens, including two juveniles and two mature adults. Six pairs of appendages, including the chelicerae and five pairs of walking legs, can be recognized on the ventral side of the prosoma. The distal-most articles of all appendages do not reach the margin of the carapace. The anterior-most pair, the chelicerae, are small relative to the walking legs (Fig. 2B–C), as they are in modern horseshoe crabs. Appendage pairs 2–5 have five articles, of which the last article comprises a hand with extended finger and an articulating second finger to form the chela. The second pair of appendages, the pedipalps, are well preserved in one individual (Fig. 2A,F). There are two distal fingers on the pedipalp, of which the movable one curves outward at the end with the relatively straight distalmost one (Fig. 2F). It worthy of note that the pedipalp of *Y. luopingensis* is almost identical to the modified male pedipalp of *Carcinoscorpius*. In *Carcinoscopius* the chela is bent forward to form a clasper (Supplementary Fig. S2), although it is not as heavily modified as in *Limulus* and *Tachypleus* (Supplementary Fig. S1). The third appendage (walking leg 2) in the specimen also has the chela bent forward into a clasper. The fourth to fifth pairs of walking legs are chelate, and similar to one another in morphology. The sixth appendages, or the pushers (Fig. 2A,D,G), are different from the anterior limbs and are like those of modern horseshoe crabs. They serve to facilitate digging as well as swimming. The pushers are large and strong, and at least five articles are recognized. The terminal article bears a whorl of four petal-like plates and a tiny chela. The chela on the last article of the pushers is much smaller than those on the four preceding pairs of walking legs. It extends well beyond the position of the petal-like plates, just as it does in extant *Limulus*. The coxa and gnathobases are observed in the juvenile individual (Fig. 2B). The gnathobases are preserved as small spines present on the edge of the coxa. Besides *Y. luopingensis*, pusher legs are also present in *Psammolimulus gottingensis* from the Middle Buntsandstein (late Early to early Middle Triassic) of Germany^18^, which is slightly older than *Y. luopingensis*.

Opisthosomal appendages include a broad plate and five smaller plate-like structures. The broad plate is interpreted as the genital operculum. The posteriormost five, referred to as the gill opercula, each bear numerous broad, flat gills on their dorsal surfaces. They form a series of numerous gill lamellae interleaved between successive opercula; thus, the term book gills. Book gills are preserved in four specimens, of which three show well-preserved details (Figs 2A and 3A–C) and one shows 3-D preservation without fine structures (Fig. 1E). Two specimens (Fig. 3A,C) show preservation of both left and right sides of all book gills. In most specimens, at least half of the anterior pairs are covered by the next pairs, but in one specimen (Fig. 3A) the book gills are well preserved without overlapping. Ventral to the gills, the plate-like opecula are observed in one specimen (Fig. 3A). Round, flap-like gill lamellae are attached to the dorsal or upper surface of the gill opercula. About 10–15 laminae per gill lamella are observed (Fig. 3E–G) and a total of at least 100 lamellae can be estimated. An genital operculum with possible genital opening is observed in one specimen (Fig. 3C), but these structures are absent in most of the available specimens. It seems likely that the absence of opercula is a preservational issue, and these structures may have been detached during taphonomic processes.

The remarkable preservation of book gills in *Yunnanolimulus luopingensis* shows the details of the respiratory organs. The overall morphology of the book gills in *Y. luopingensis* closely resembles that of extant horseshoe crabs, i.e. *Trachypleus tridentatus* ^28^ (Fig. 3D). The number of gill lamellae in *Y. luopingensis* is similar to that of the instar stage 9 in extant *Limulus polyphemus* ^29^, which have a prosomal width of 28 mm, comparable to that of *Y. luopingensis*. Previously reported examples of book gills in fossil horseshoe crabs include the Palaeozoic *Paleolimulus signatus* ^4^ and the Cretaceous *Tachypleus syriacus* ^20^. Although the book gills of *Paleolimulus signatus* are well preserved and perfect for comparison with those of *Y. luopingensis*, no detail are available in the description^4^ and further work is needed.

Setal preservation has not been documented previously in any fossil horseshoe crab. Fortunately, in one specimen of *Yunnanolimulus luopingensis*, fine setae are observed along the margin of the movable spines and the telson (Fig. 4A,B,D). The setae on both sides of the telson are dense and well preserved, with a maximum length of 0.5 mm, whereas those on the movable spines are less distinct and merely recognizable. Extant horseshoe crabs also bear fine setae on the margins of movable spines, opisthosoma, and telson. In particular, modern *Carcinoscorpius* has extremely robust, dense setae around the movable spines and telson insertion, which is similar to that of *Y. luopingensis*.

**Figure 5:**
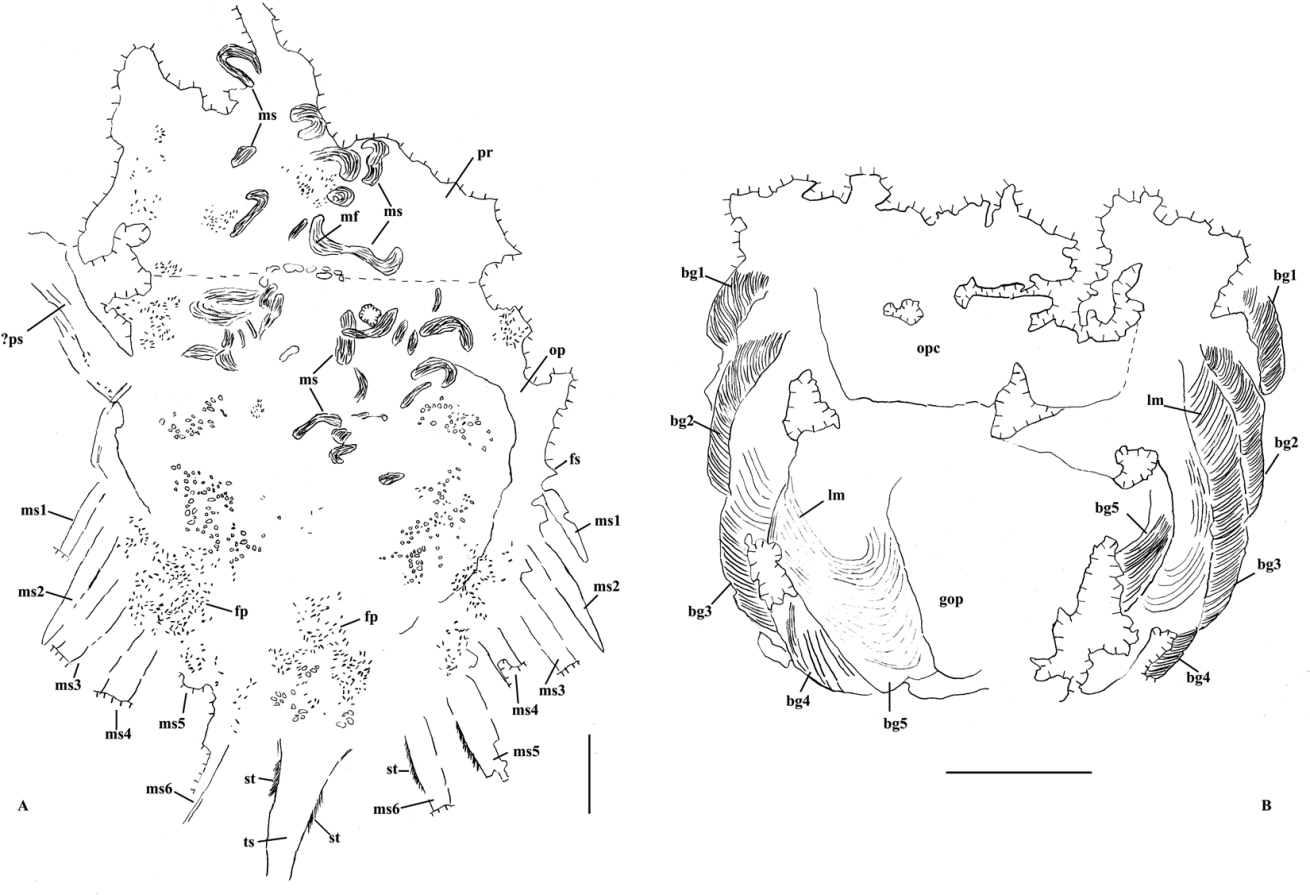
Interpretive drawings of selected specimens showing well preserved book gills, muscles, and setae. (**A**) LPI-31945, (**B**), LPI-40709. The following abbreviations are used: bg, book gills; fp, faecal pellets; fs, fixed spine; gop, gill opecula; lm, lamellae; mf, muscle fibre; ms, movable spines; op, opisthosoma; opc: genital operculum; pr, prosoma;?ps,?pusher; st, setae; ts, telson. Dash line in (**A**) indicates possible place of hinge line. Scale bars: 10 mm in (**A**); 5 mm in (**B**).

Possible muscle structures are observed in the specimen with preserved setae (Fig. 4A,C,E). Also distinct in this specimen are clusters of small round, elliptical and cylindrical pellet-like structures (Figs 4A and 5A). The muscle structures are concentrated mostly along the axis of the body, especially at the prosoma-opisthosoma boundary. The distribution pattern indicates thasome of the muscles are detached and are not all *in situ*. At least two types of musculature can be recognized, based upon the orientation of the muscles relative to the structures to which they attach, the circular muscles (Fig. 4E) and axial longitudinal muscles (Fig. 4C). The overall shape of some circular muscles is similar to that of the book gills. But the size of the circular muscles is much smaller, only 2–3 mm in diameter. The surface of the muscles shows lineated transverse structures (Fig. 4B–C), which are comparable in size and overall arrangement to the muscle fibres of modern horseshoe crabs^30^. Preservation of muscles in fossil horseshoe crabs is very rare. Most documented examples are fragmentary, with the exception of the well-preserved musculature of *Mesolimulus walchi* from the Upper Jurassic (Kimmeridgian) Plattenkalk of Nusplingen, Germany^21^ and *Tachypleus syriacus* from the Cretaceous of Lebanon^20^. As shown by *M. walchi*, there is no significant muscle difference between Mesozoic and extant horseshoe crabs.

## Discussion

The discovery of exceptionally preserved soft anatomy as well as appendages in *Yunnanolimulus luopingensis*, provides key information on horseshoe crab phylogeny and lifestyle. Although rarely preserved, appendages are known for each of the major horseshoe crab clades. Based on the limb data, the Palaeozoic *Offacolus*, *Dibasterium*, *Weinbergina* and *Venustulus* are placed in the euchelicerate stem. The occurrence of book gills in fossil eurypterids suggests that expansive book gills originated before the divergence of xiphosurans and eurypterids^31^. Large opercula preserved in stem euchelicerates such as *Weinbergina* ^9^, *Offacolus* ^11^, *Dibasterium* ^13^ and in eurypterids^7^ indicates that large opercula must have evolved prior to the origins of xiphosurids.

As mentioned above, *Carcinoscorpius*-type claspers already occur on the first and second walking legs in male individuals of *Y. luopingensis*. This is important, as in both *Carcinoscorpius* and *Tachypleus* the first and second walking legs form claspers in males, while only the first walking leg is modified into a clasper in *Limulus*. Given that the claspers of *Yunnanolimulus* and *Carcinoscorpius* are more rudimentary than those of *Tachypleus* and *Limulus*, this would suggest that the bulbous claspers in *Tachypleus* and *Limulus* are derived, and that the plesiomorphic condition is to have both the first and second walking legs coopted for use as claspers in males. Furthmore, the similarity of both prosomal legs and opisthosomal limbs between *Yunnanolimulus luopingensis* and extant horseshoe crabs suggests that appendage synapomorphies of fossil and extant horseshoe crabs arose at least in the Triassic. This is consistent with the results of a recent phylogenetic analysis^8^ in which *Y. luopingensis* lies stemward of the extant clade that includes all modern representatives.

The close resemblance between the Middle Triassic *Yunnanolimulus luopingensis* and extant horseshoe crabs, both in exoskeleton and soft tissues, supports similar behaviours, such as burrowing, swimming, righting, and mating. *Y. luopingensis* probably burrowed like its modern relatives, pushing the body forward and downward through the soft surface of the substrate using the pusher legs bearing four leaf-like processes. It seems reasonable to hypothesize that *Y. luopingensis* swam inverted, using its legs and gills for propulsion, based upon the possession of very similar morphology to extant horseshoe crabs. Like those of extant horseshoe crabs, the gills of *Y. luopingensis* most likely provided a continuous flow of water over the respiratory surfaces of the book gills during life. During swimming, the animal achieved propulsion by systematic movements of walking legs, operculum, and gills^32^. The modern-style walking legs and telson might have facilitated the righting process. The similarity of setal arrangement, where preserved on the margin of the telson and movable spines in *Y. luopingensis* and extant horseshoe crabs, possibly indicates the same function, namely as sensory receptors of physical stimulation^33^ or as mechanoreceptors that aid in the burrowing and burying process^34^. Similar copulation behaviour to extant horseshoe crabs might have occurred since the Middle Triassic, as indicated by the almost identical appendage II and appendage III between male *Y. luopingensis* and extant *Carcinoscorpius*.

The exceptional preservation of appendages as well as some muscles, gills, and setae in *Yunnanolimulus luopingensis* also reveals a striking degree of morphological and ecological stasis in the evolutionary history of marine horseshoe crabs. Their basic body plan, both the exoskeleton and soft tissues, has been fundamentally conserved since at least the Early Mesozoic, implying an evolutionary conservatism of marine horseshoe crabs for over 240 million years. The factors responsible for such morphological and ecological stasis may result from habitat stability. Most horseshoe crabs inhabit marginal environments, which have been remarkably stable throughout geological history^35^. In contrast, non-marine horseshoe crab taxa demonstrate significant innovations in exoskeletal morphology^8^. Whether this innovation is confined to the exoskeleton or is associated with internal anatomical changes remains unknown. Further discoveries of soft tissues in fossils of non-marine horseshoe crabs would shed new light on their evolutionary pathway.

## Methods

Fossil materials of *Yunnanolimulus luopingensis* were obtained by splitting micritic limestone in the field. Further preparation was carried out in the laboratory with sharp blades under a binocular microscope. Fossils were photographed with a Canon Mark II Camera with an EF 100mm f/2.8 L IS USM close-up lens under incident light. Enlargements of details at mm scale were photographed with a Leica DFC295 camera mounted on a Leica M125 photo-micrographic system under fibre-optic lights. The images were processed in Adobe Photoshop CS3. Based on the observation of specimens with preserved soft-tissues, artistic reconstruction of *Y. luopingensis* is made in both dorsal and ventral views (Supplementary Fig. S3).

## Electronic supplementary material

Supplementary Figures S1-S3
