# Supplementary material for: Exceptional appendage and soft-tissue preservation in a Middle Triassic horseshoe crab from SW China
Source: Sci Rep. 2017 Oct 26;7:14112. doi: 10.1038/s41598-017-13319-x (PMC5658423; doi:10.1038/s41598-017-13319-x)
Supplement: Supplementary file 1 — Supplementary Figures S1-S3 [file 41598_2017_13319_MOESM1_ESM.pdf]

Supplementary Information

Title: Exceptional appendage and soft-tissue preservation in a Middle Triassic horseshoe crab from SW China

Authors: Shixue Hu[1,2]*, Qiyue Zhang[1,2], Rodney M. Feldmann[3], Michael J. Benton[4], Carrie E. Schweitzer[5], Jinyuan Huang[1,2], Wen Wen[1,2], Changyong Zhou[1,2],Tao Xie[1,2], Tao Lü[1,2], Shuigen Hong[6]

1 Chengdu Center of China Geological Survey, Chengdu 610081, China

2 Chengdu Insitute of Geology and Mineral Resources, Chengdu 610081, China

3 Department of Geology, Kent State University, Kent, OH 44242, USA

4 School of Earth Sciences, University of Bristol, Bristol BS8 1RJ, UK

5 Department of Geology, Kent State University at Stark, 6000 Frank Avenue NW, North Canton, OH 44720, USA

6 Institute of Neuroscience, Xiamen University, Ximang 361005, China

* Correspondence and requests for materials toShixue Hu (hushixue@126.com)

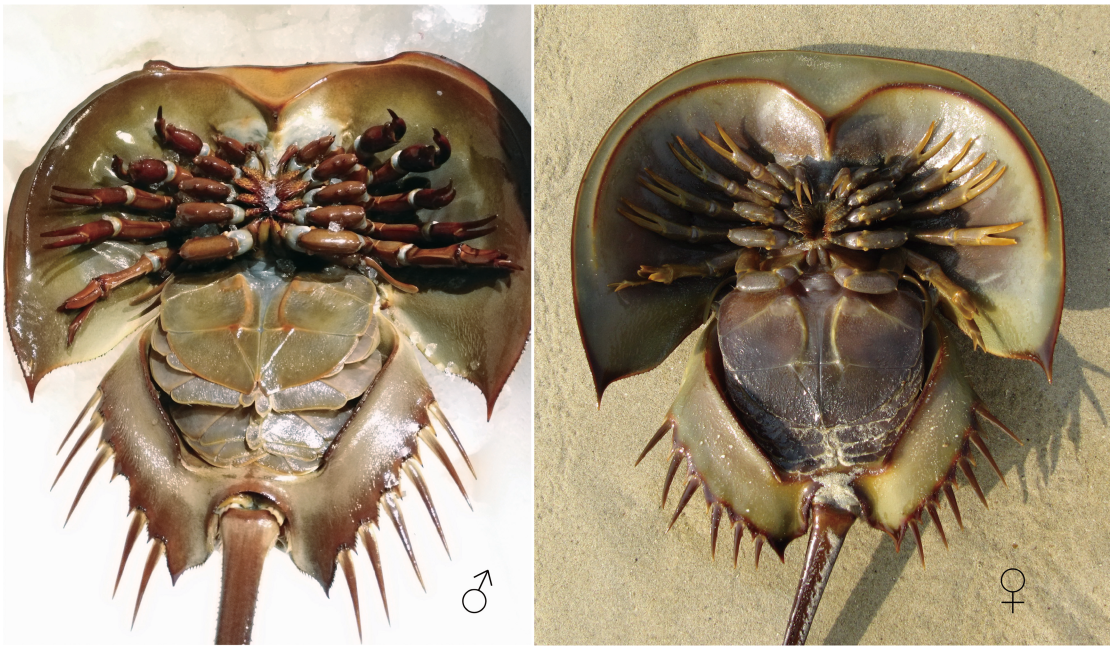

Supplementary Figure S1 Ventral view of male (left) and female (right) of extant *Tachypleus tridentatus.*

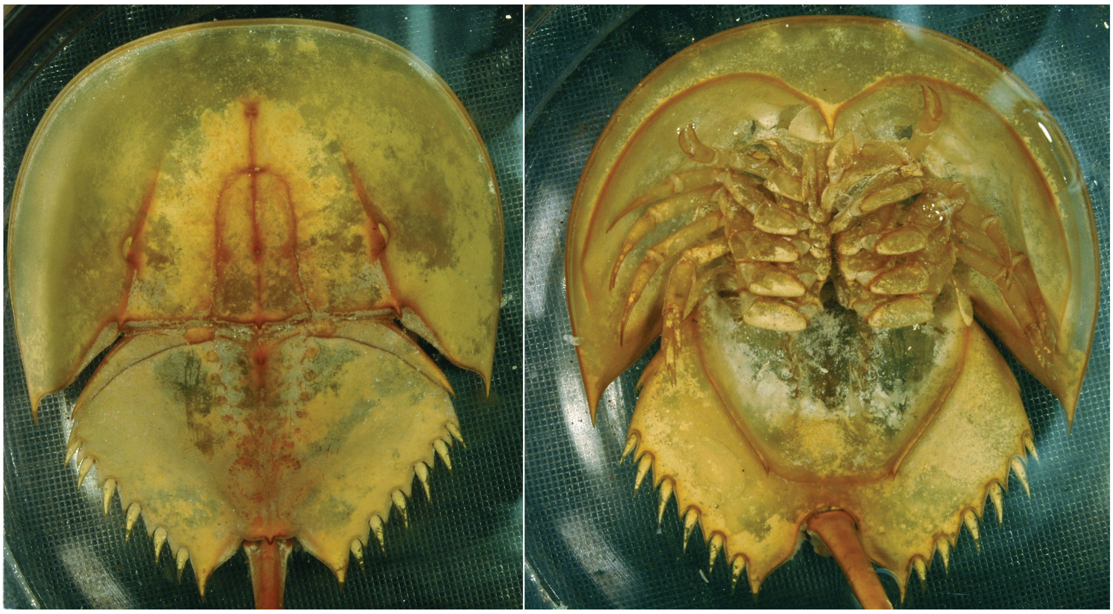

Supplementary Figure S2 Dorasl and ventral view of extant male *Carcinoscorpius rotundicauda*. Opisthosomal appendages and related tissues were removed.

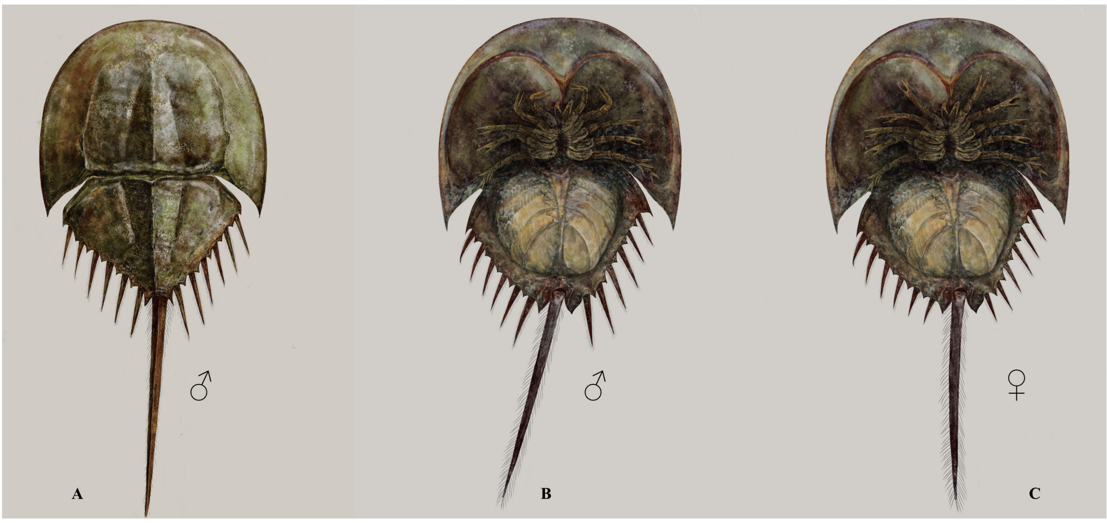

Supplementary Figure S3 Reconstruction of *Yunnanolimulus luopingensis*. A, male individual in dorsal view; B, male individual in ventral view; C, female individual in ventral view. The opercula are not shown in ventral view in order to present the gill lamellae. Illustration by Qingtao Chen.
